# Anti-complement Treatment for Paroxysmal Nocturnal Hemoglobinuria: Time for Proximal Complement Inhibition? A Position Paper From the SAAWP of the EBMT

**DOI:** 10.3389/fimmu.2019.01157

**Published:** 2019-06-14

**Authors:** Antonio M. Risitano, Serena Marotta, Patrizia Ricci, Luana Marano, Camilla Frieri, Fabiana Cacace, Michela Sica, Austin Kulasekararaj, Rodrigo T. Calado, Phillip Scheinberg, Rosario Notaro, Regis Peffault de Latour

**Affiliations:** ^1^Department of Clinical Medicine and Surgery, Federico II University of Naples, Naples, Italy; ^2^Severe Aplastic Anemia Working Party of the European Group for Blood and Marrow Transplantation, Leiden, Netherlands; ^3^Laboratory of Cancer Genetics and Gene Transfer, Core Research Laboratory - Istituto per lo Studio, la Prevenzione e la Rete Oncologica (ISPRO), Florence, Italy; ^4^Department of Haematological Medicine, King's College Hospital, National Institute of Health Research/Wellcome King's Clinical Research Facility, London, United Kingdom; ^5^Department of Hematology and Oncology, University of São Paulo at Ribeirão Preto School of Medicine, São Paulo, Brazil; ^6^Division of Hematology, Hospital A Beneficência Portuguesa, São Paulo, Brazil; ^7^French Reference Center for Aplastic Anemia and Paroxysmal Nocturnal Hemoglobinuria, Saint Louis Hospital and University Paris Diderot, Paris, France

**Keywords:** paroxysmal nocturnal hemoglobinuria, intravascular hemolysis, extravascular hemolysis, complement inhibition, eculizumab, ravulizumab, compstatin

## Abstract

The treatment of paroxysmal nocturnal hemoglobinuria has been revolutionized by the introduction of the anti-C5 agent eculizumab; however, eculizumab is not the cure for Paroxysmal nocturnal hemoglobinuria (PNH), and room for improvement remains. Indeed, the hematological benefit during eculizumab treatment for PNH is very heterogeneous among patients, and different response categories can be identified. Complete normalization of hemoglobin (complete and major hematological response), is seen in no more than one third of patients, while the remaining continue to experience some degree of anemia (good and partial hematological responses), in some cases requiring regular red blood cell transfusions (minor hematological response). Different factors contribute to residual anemia during eculizumab treatment: underlying bone marrow dysfunction, residual intravascular hemolysis and the emergence of C3-mediated extravascular hemolysis. These two latter pathogenic mechanisms are the target of novel strategies of anti-complement treatments, which can be split into terminal and proximal complement inhibitors. Many novel terminal complement inhibitors are now in clinical development: they all target C5 (as eculizumab), potentially paralleling the efficacy and safety profile of eculizumab. Possible advantages over eculizumab are long-lasting activity and subcutaneous self-administration. However, novel anti-C5 agents do not improve hematological response to eculizumab, even if some seem associated with a lower risk of breakthrough hemolysis caused by pharmacokinetic reasons (it remains unclear whether more effective inhibition of C5 is possible and clinically beneficial). Indeed, proximal inhibitors are designed to interfere with early phases of complement activation, eventually preventing C3-mediated extravascular hemolysis in addition to intravascular hemolysis. At the moment there are three strategies of proximal complement inhibition: anti-C3 agents, anti-factor D agents and anti-factor B agents. These agents are available either subcutaneously or orally, and have been investigated in monotherapy or in association with eculizumab in PNH patients. Preliminary data clearly demonstrate that proximal complement inhibition is pharmacologically feasible and apparently safe, and may drastically improve the hematological response to complement inhibition in PNH. Indeed, we envision a new scenario of therapeutic complement inhibition, where proximal inhibitors (either anti-C3, anti-FD or anti-FB) may prove effective for the treatment of PNH, either in monotherapy or in combination with anti-C5 agents, eventually leading to drastic improvement of hematological response.

## Introduction

Paroxysmal nocturnal hemoglobinuria (PNH) is a rare hematological disorder characterized by complement-mediated intravascular hemolysis, bone marrow failure, and severe thrombophilia (1). PNH is due to the expansion of hematopoietic stem cells (HSCs) bearing somatic loss-of-function mutations in the phosphatidylinositol N-acetylglucosaminyltransferase subunit A (*PIGA*) gene (2–4). The *PIGA* genetic lesion impairs the biosynthesis of the glycosylphosphatidylinositol (GPI) anchor, and as a consequence all GPI-linked proteins are not expressed on affected HSC and their mature progeny blood cells (5–8). Among the missing GPI-linked proteins, the lack of the two complement inhibitors CD55 (9–11) and CD59 (12, 13) makes erythrocytes susceptible to complement lysis. However, the expansion of the mutated HSCs, which is essential to develop the disease, presumes a concomitant immune-mediated damage of normal hematopoiesis, from which *PIGA* mutated HSCs are spared (14–16). Treatment options for hemolytic PNH remained limited and often inadequate until eculizumab became available, a humanized monoclonal antibody (mAb) targeting the component 5 (C5) of the complement cascade (17). Indeed, by disabling the complement cascade at the level of the terminal complement step (i.e., membrane attack complex—MAC—formation) eculizumab prevents the lysis of PNH erythrocytes, which cannot properly curb complement activation on their surface (1). The efficacy of eculizumab in PNH patients was first demonstrated in a pilot study from the United Kingdom, which showed robust inhibition of complement-mediated intravascular hemolysis (18). Two subsequent large international phase III randomized studies demonstrated that eculizumab prevents intravascular hemolysis in PNH, eventually leading to hemoglobin stabilization, reduction/eradication of red blood cell transfusions, and resolution of most disease-related symptoms (19, 20). These data were confirmed in longer follow up analyses, which showed further hematological improvement on continuous maintenance treatment with eculizumab, with no safety concerns (21). Notably, eculizumab also reduced the thromboembolic risk (22), the most serious complication in PNH, thereby impacting on the disease course, morbidity and long-term survival. Indeed, with the caveat of the relatively short follow up, two independent studies have shown that PNH patients receiving continuous treatment with eculizumab have a 5 year survival >90% (23, 24). These survival rates appear superior to the rate reported on the natural history of PNH (25–27), elegantly shown in a retrospective comparison between eculizumab-treated patients and historical controls (24). Thus, after its approval in 2007, eculizumab is considered to this date the standard of care for PNH patients with hemolytic disease or thromboembolic complications. Despite the fact that eculizumab was a breakthrough therapy for PNH, recent efforts are aimed to further improve this current standard in PNH. In this manuscript, we review current gaps in anti-complement treatment for PNH, eventually setting the goals for future complement inhibitors in development for PNH.

## Hematological Response in Pnh During Eculizumab

Anti-complement treatment with the anti-C5 monoclonal antibody eculizumab results in sustained inhibition of complement-mediated hemolysis in almost all PNH patients (19, 20); however, in the registration trials the endpoints were mostly set on transfusion independence and reduction of hemolysis, assessed by LDH. Although hemoglobin stabilization was achieved in most patients (including transfusion independent patients), many exhibited significant improvement in hemoglobin level but still remained variably anemic (19–21). However, well-defined response categories had not been established. In 2009, we empirically classified hematological response in PNH patients on eculizumab as follows: (i) optimal response (no transfusions, hemoglobin stable >11 g/dL); (ii) good response (no transfusion, hemoglobin ranging between 8 and 11 g/dL); (iii) partial response (still transfused, but with transfusion requirement reduced by at least 50%); (iv) minor response (transfusion requirement unchanged, or reduced by <50%) (28). In this study, we showed that no more than one third of PNH patients on eculizumab achieved normal hemoglobin values, leading us to investigate possible explanations for this limited and less than anticipated hematological benefit. Ten years later, additional long-term data have confirmed that, despite of the overall sustained efficacy and improved survival under eculizumab treatment, hematological benefit from eculizumab can be variable (21, 23, 24); thus, in addition to transfusion independence, hemoglobin normalization appears to be a discrete endpoint which can be used to characterize hematological response. Thus, considering that nowadays most new PNH patients start anti-complement therapies before receiving many transfusions, the following response categories can be proposed (Table 1): (i) complete response (no transfusion with normal hemoglobin stable and no evidence of hemolysis); (ii) major response (no transfusion with normal hemoglobin, with evidence of intravascular or extravascular hemolysis); (iii) good response (no transfusion, with persistent chronic mild anemia or evidence of residual intravascular hemolysis); (iv) partial response (persistent chronic moderate anemia and/or occasional red blood cell transfusions); (v) minor response (regular red blood cell transfusions); (vi) no response (regular and frequent red blood cell transfusions). For PNH patients with documented history of regular blood cell transfusions before starting eculizumab, these two latter hematological response categories may also be defined based on the reduction of the transfusion burden: patients with reduction ≥50% may be classified as minor responders, whereas those with reduction <50% may be classified as non-responders. Patients with suboptimal hematological response can be further distinguished based on the evidence of persistent intravascular hemolysis (based on LDH ≤ 1.5 or >1.5 ULN). It is important to emphasize that no hematological response does not necessarily mean no clinical benefit from eculizumab (see the effect on thromboembolisms in PNH, eculizumab treatment section), or, in the future, to other anti-complement agents: it is a very useful tool to better understand the reasons underlying unsatisfactory hematological benefit, eventually driving therapeutic decisions (e.g., modified treatment schedules, or addition/switch to different inhibitors). Indeed, at the moment there is no clear evidence about the possible impact (if any) of a suboptimal hematological response to eculizumab on its prevention of thrombosis, and on its long-term survival benefit.

**Table 1:** Tentative classification of hematological response to anti-complement agents in PNH.

**Response category**	**Red blood cell transfusions**	**Hemoglobin level**	LDH level^*^^‡^	ARC^*^
Complete response	None	≥12 g/dL	≤1.5x ULN	***and*** ≤ 150,000/μL^§^
Major response	None	≥12 g/dL	>1.5x ULN	***or*** >150,000/μL^§^
Good response	None	≥10 and <12 g/dL	A. ≤ 1.5x ULN	Rule out bone marrow failure^°^
			B. >1.5x ULN	
Partial response	None or occasional (≤ 2 every 6 months)	≥8 and <10 g/dL	A. ≤ 1.5x ULN B. >1.5x ULN	Rule out bone marrow failure^°^
Minor response^#^	None or occasional	<8 g/dL		Rule out bone marrow failure^°^
	(≤ 2 every 6 months)		A. ≤ 1.5x ULN	
	Regular (3–6 every 6 months)	<10 g/dL	B. >1.5x ULN	
	Reduction by ≥50%^∧^	<10 g/dL		
No response^#^	Regular (>6 every 6 months)	<10 g/dL	A. ≤ 1.5x ULN	Rule out bone marrow failure^°^
			B. >1.5x ULN	

LDH, lactate dehydrogenase; ULN, upper limit of the normal; ARC: absolute reticulocyte count.

* Response categories are mostly based on red blood cell transfusion and hemoglobin level, but LDH and ARC serve as ancillary indicators to discriminate between complete and major response, as well as within suboptimal response categories.

‡ A. and B. indicate subcategories without or with residual significant intravascular hemolysis, respectively.

§ To rule out increased erythropoietic response to compensate ongoing hemolysis; the value of 150,000/μL is a tentative index based on 1.5x ULN (which in most laboratories is set at 100,000/μL).

° To assess the relative contribution of the degree of bone marrow failure to any response less than complete: a value of ARC below 60,000/μl could be a tentative index to establish such a contribution; bone marrow investigation may be appropriate.

∧ For patients with previous transfusion history (with a pre-treatment follow up of at least 6 months).

# *For patients who do not accept red blood cell transfusions, minor response can be defined based on hemoglobin level ≥6 and <8 g/dL, and no response based on hemoglobin <6 g/dL. All hemoglobin, LDH and ARC values should be assessed based on the median value over a period of 6 months*.

Intrinsic resistance to eculizumab has been reported, albeit very rare, and it is associated with inherited polymorphism of C5 which prevents eculizumab binding (29); but, in all other patients, eculizumab is biologically active and reduces intravascular hemolysis with unpredictable hematological benefits. There are several factors which contribute to such heterogeneity which are discussed herein (Table 2) (30). Bone marrow function is the most obvious contributor, since immune-mediated bone marrow failure is a key element of the pathophysiology of PNH (14). In this context, it is worth mentioning that impaired bone marrow function may become clinically meaningful even without overt aplastic anemia, given the lack of a compensatory increase in erythropoiesis with continuous hemolysis. Second is the efficacy of the inhibition of intravascular hemolysis; as discussed below, residual intravascular hemolysis is detectable in most PNH patients on eculizumab, and may become clinically relevant in specific conditions. And thirdly is the occurrence of C3-mediated extravascular hemolysis (28); this novel and unanticipated mechanism of hemolysis is mechanistically associated with anti-C5 therapies. Since many of these factors may contribute to the ultimate hematological response in PNH patients, it is essential that their contribution is adequately investigated during eculizumab therapy (31).

**Table 2:** Reasons for inadequate hematological response to eculizumab and possible actions.

**Reason**	**Cause**	**Prevalence**	**Mechanism**	**Clinical impact on hematological response**	**Corrective action**
Intravascular hemolysis	Inherited C5 variants	Ultra-rare (<1%, usually in Japanese patients)	Intrinsic resistance due to impaired binding of eculizumab (and of ALXN1210)	Minimal (but very significant for the few patients for whom there is no available treatment)	Switch to other investigational agents (mostly alternative C5 inhibitors)
	Recurrent pharmacokinetic breakthrough	10–15% of patients	Inadequate plasma level of eculizumab	Significant	Decrease interval of dosing (10–12 days) or increase dose of eculizumab (1,200 mg), or consider novel investigational agents
	Sporadic pharmacodynamics breakthrough	May occur in any patients	Massive complement activation due to concomitant clinical events	Minimal	None (treat the underlying cause)
Extravascular hemolysis	C3-mediated extravascular hemolysis	25–50% of patients (even more considering subclinical events)	Persistent uncontrolled activation of proximal complement, leading to C3-fragment opsonization of PNH red blood cells and subsequent removal by professional hepato-splenic phagocytes	Very significant	Consider employing investigational proximal inhibitors of the complement
Bone marrow disorders	Bone marrow failure	10–35% (depending also on initial patient selection)	Inadequate production of red blood cells	Significant	Treat underlying aplastic anemia with either immunosuppression or bone marrow transplantation
	Clonal evolution to myeloid malignancies	1–5%	Additional stochastic somatic mutations	Relevant	Treat the myeloid malignancy

### Thromboembolisms in PNH During Eculizumab Treatment

The clinical benefit of eculizumab in PNH goes beyond the inhibition of intravascular hemolysis and possible hemoglobin stabilization; indeed, another consequence of therapeutic complement blockade is the prevention of thromboembolism. In the registration trials, the rate of thromboembolism during eculizumab treatment was reduced by 85% as compared with the pretreatment rate in the same patients (from 7.37 to 1.07 events/100 patient-years) (22). This effect was demonstrated even in patients already on anti-thrombotic treatment (mostly patients with previous thromboembolic events, thus the population at the highest risk of new thromboembolisms), with rate of thromboembolism reduced from 10.61 to 0.62 events/100 patient-years with eculizumab treatment (22). Nevertheless, albeit rarely, thromboembolic events may appear even during eculizumab treatment (23, 24); in analogy with intravascular hemolysis, these events may be defined as “*breakthrough thromboembolisms.”* It is not entirely clear how eculizumab mechanistically prevents thromboembolism in PNH (e.g., direct inhibition of complement-mediated activation on PNH platelets, or indirect effect due to reduced intravascular hemolysis). Thus, the possible relationship of breakthrough thromboembolisms with suboptimal complement blockade and/or residual intravascular hemolysis, as well as the contribution of non-PNH related factors, need to be assessed individually in each patient, and further mechanistic investigations would be welcome, including assessment of possible biomarkers of such a risk.

### Bone Marrow Function in PNH

As stated earlier, PNH is not simply a hemolytic anemia; indeed, a bone marrow disorder is always assumed to allow for the expansion of *PIGA* mutated HSCs (14, 15), which may appear as immune-mediated aplastic anemia (AA) (16). About 40% of PNH patients develop meaningful AA during their disease course (27); but even in milder forms, immune-mediated bone marrow failure may contribute to cytopenias, including anemia. The treatment of AA in the context of PNH is out of the scope of this review and it will not be discussed in detail; however, two important points are worth highlighting. First, the presence of a PNH clone in the context of severe AA (SAA) does not change the management of SAA: patients younger than 40 years with a matched related donor should proceed to bone marrow transplantation (BMT) (32), whereas immunosuppression (horse anti-thymocyte globulin and cyclosporine) is the preferred first-line treatment for patients older than 40 years, or lacking a matched related donor (33, 34). The addition of the thrombopoietin-mimetic agent eltrombopag in combination with standard immunosuppression seems very promising and has been approved in the U.S. as first-line (35). Currently underway is a phase III randomized study conducted by the Severe Aplastic Anemia Working Party of the EBMT comparing horse anti-thymocyte globulin and cyclosporine ± eltrombopag (36). Second, more severe forms of AA in most cases represent a contraindication to anti-complement treatment, which does not reverse the marrow failure component and should be reserved for patients with more adequate bone marrow function (i.e., no severe neutropenia or thrombocytopenia, and compensatory reticulocytosis adequate to hemoglobin levels). Nevertheless, sometimes clinically significant hemolytic anemia and bone marrow failure may appear concomitantly in the same patient, or, more commonly, may develop at different times during the disease course. In these circumstances, the indication for anti-complement treatment should be evaluated individually, since in selected cases complement inhibition can be clinically effective in AA-PNH and could be used concomitantly or sequentially to standard immunosuppression (eculizumab does not worsen other cytopenias) (24). Thus, continuous evaluation of bone marrow function is mandatory in all hemolytic PNH patients receiving anti-complement treatment. In addition, similarly to AA and other bone marrow failure syndromes, PNH harbors a risk, albeit a low one, of evolution into myeloid malignancies such as myelodysplastic syndromes and acute leukemia (27). If immune-mediated bone marrow failure develops in the context of a hemolytic PNH, concomitant or sequential treatment with immunosuppression and anti-complement agents may be considered (37–39). Other non-transplant therapies in AA that directly stimulate HSC (eltrombopag) are making a positive impact in some patients (35, 40, 41); however, the use of eltrombopag in AA/PNH will require specific investigations for the risk of expansion of the PNH clone. Since the complement cascade is not involved in the pathophysiology of immune-mediated bone marrow failure, the improvement of anti-complement therapeutic strategies is not expected to improve the treatment of marrow disorder underlying PNH.

## Unmet Clinical Needs in Anti-Complement Treatment For Pnh

### Intravascular Hemolysis in PNH During Eculizumab Treatment

As initially shown in the registration trials (19, 20), treatment with eculizumab results in sustained control of complement-mediated intravascular hemolysis in all PNH patients. Nevertheless, using lactate dehydrogenase (LDH) as the best biomarker of hemolysis, the vast majority of patients continue to show slightly increased LDH, usually ranging between 1- and 1.5 times the upper limit of normal (ULN), in addition to persistently undetectable haptoglobin (19–21). This observation has raised the notion that minimal, residual intravascular hemolysis is common during eculizumab treatment, even if it is clinically not relevant in the majority of patients. The reasons for this less than optimal complement inhibition *in vivo* have not been fully elucidated. Some of us have demonstrated that residual complement activity (assessed by a functional assay measuring the 50% of complement hemolytic activity—CH50) can be detected in several PNH patients on eculizumab, and it correlates with plasma LDH levels (42). This residual complement activity (CH50 >10%) also correlates with low plasma levels of free eculizumab, eventually suggesting that suboptimal (or even partial) C5 blockade may occur due to subtherapeutic plasma levels of eculizumab (42). Nevertheless, in this broad PNH population suboptimal C5 blockade does not seem to be associated with lower hemoglobin levels (in the sense that most patients remain anemic irrespective of full C5 blockade), eventually arguing against a predictive clinical value for LDH, CH50, or any other laboratory measurement in the context of eculizumab treatment. On the other hand, in a few patients meaningful laboratory findings (i.e., LDH >1.5x ULN) and clinical hemolysis can be detected during eculizumab treatment; in this condition CH50, or other more sophisticated functional complement assays, may confirm partial C5 blockade, eventually further justifying specific therapeutic intervention (43).

### Breakthrough Hemolysis: Pharmacokinetics vs. Pharmacodynamics

The reappearance of hemolysis in a PNH patient on eculizumab has been described as “breakthrough hemolysis.” There is no formal definition for this condition, but it seems very important to have it, since it will be eventually exploited as an endpoint in future trials investigating novel anti-complement agents, and its elimination may represent a clinical goal for any new therapy for PNH. Clinical breakthrough hemolysis is identified by the appearance of clinical symptoms such as painful hemolytic crises and dark urines (somehow subjective), associated with a rise in LDH and a drop in hemoglobin. Sometimes hemolysis may be evident just by laboratory data (i.e., LDH or hemoglobin) and hemoglobinuria: this may be referred to as subclinical breakthrough hemolysis. More robust definitions for clinical and subclinical breakthrough are needed, and we suggest the following classification (Table 3): breakthrough hemolysis should be individualized to each patient's steady-state LDH, and significant hemoglobin drop in a given period or clinically meaningful signs or symptoms of hemolysis should be acknowledged as a clinical event. For instance, ***clinical breakthrough hemolysis***may be defined by a hemoglobin drop ≥ 2 g/dL within 2 weeks or the development of clinical signs or symptoms of hemolysis, in combination with laboratory demonstration of increased intravascular hemolysis (LDH >1.5x ULN, increased as compared to the latest available value). In contrast, the isolated laboratory evidence of increased intravascular hemolysis (>1.5x ULN, increased by at least 50% as compared to the latest available value, or a discrete hemoglobinuria), without meaningful drop in hemoglobin (<2 g/dL) and without other clinical signs or symptoms of hemolysis, may be defined ***subclinicalbreakthrough hemolysis***.

**Table 3:** Definition of clinical and subclinical *breakthrough* hemolysis during eculizumab treatment for PNH.

	**Clinical criteria**		**Laboratory criteria**
	**Hemoglobin level**	**Sign or symptoms**	**LDH level**
Clinical breakthrough^*^	Drop ≥2 g/dL (compared to the latest assessment, within 15 days)	Gross hemoglobinuria, painful crisis, dysphagia or any other significant clinical finding	>1.5x ULN (and increased as compared to the steady-state)
Subclinical breakthrough	Drop <2 g/dL (compared to previous assessment, within 15 days)	No clinical symptom or sign, except moderate hemoglobinuria	>1.5x ULN (and increased by at least 50% as compared to the steady-state)

LDH, lactate dehydrogenase; ULN, upper limit of the normal.

* *The breakthrough is defined clinical if either one of the two clinical criteria is demonstrated, in presence of the laboratory evidence of intravascular hemolysis (LDH level)*.

**Figure 1:**
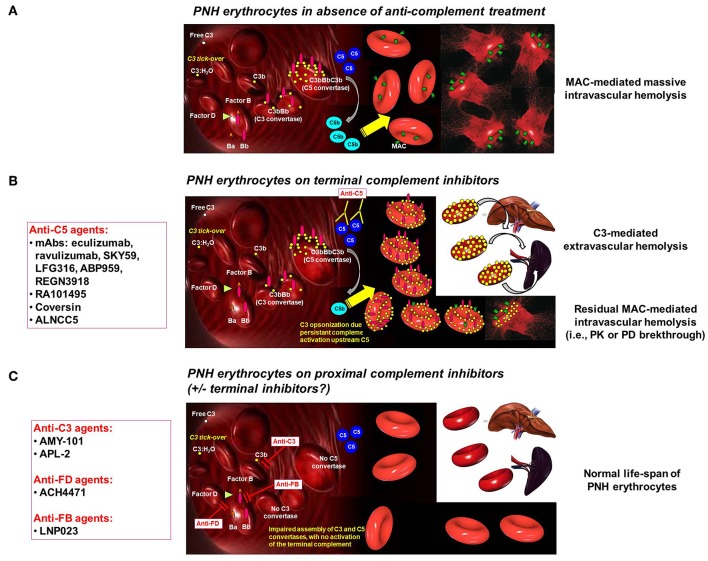
**Complement activation on PNH erythrocytes**. **(A) *PNH erythrocytes inabsence of anti-complement treatment***. The complement system may activate due to different triggers through the alternative, classical and mannose/lectin pathway. Spontaneous C3 tick-over continuously generates low-grade activation of the alternative pathway in the fluid phase and possible binding of activated C3 fragments on erythrocytes. Due to the lack of CD55, this leads on PNH erythrocytes to the generation of C3 convertase, with further generation of C3b, which eventually leads to the assembly of C5 convertase. Then, the terminal pathway of the complement cascade is activated, with the generation of the MAC, eventually leading to lysis of PNH erythrocytes lacking CD59. **(B) *PNH erythrocytes on terminal complement inhibitors***. Terminal complement inhibitors (i.e., anti-C5 agents) prevent the cleavage of C5 into C5a and C5b, thereby disabling the formation of the MAC. Thus, PNH erythrocytes are largely protected from intravascular lysis. Nevertheless, early phases of surface complement activation remain uncontrolled on PNH erythrocytes due to the lack of CD55; thus, continuous low-grade activation continues leads to opsonization of PNH erythrocytes with C3 fragments. This excess of C3 generates high-affinity C5 convertases, which may account for residual intravascular hemolysis due to pharmacodynamic breakthrough (in addition to possible pharmacokinetic breakthrough due to sub-therapeutic plasma leven of anti-C5 agent). Moreover, C3 opsonization leads to extravascular hemolysis due to C3-specific receptors expressed on professional macrophages in the liver and in the spleen. **(C) *PNH erythrocytes on proximal complement inhibitors(*± *terminal complement inhibitors)***. Proximal complement inhibitors intercept complement activation at the level of its key component C3 (i.e., anti-C3 agents), or even upstream at the level of initial activation of the alternative pathway (i.e., anti-FD and anti-FB agents). All these agents prevent early activation of complement on the surface of PNH erythrocytes, counterbalancing the deficiency of the complement regulators CD55 and CD59. Based on theoretical assumptions and *in vitro* data, proximal complement inhibitors prevent C3 opsonization, thereby preventing C3-mediated extravascular hemolysis. However, by disabling early surface complement activation, proximal complement inhibitors should also prevent intravascular hemolysis. While preliminary clinical data already confirmed that proximal complement inhibitors prevent C3-mediated extravascular hemolysis, ongoing investigation will make clear whether they can adequately prevent intravascular hemolysis even in the absence of terminal inhibitors (as already documented *in vitro*).

Irrespective of the reliability of the current definition, breakthrough hemolysis has been described since the very first experiences with eculizumab (19, 20). Mechanistically, breakthrough hemolysis obviously results from a temporary decrease in complement inhibition, leading to some perturbation of the previous steady-state. Indeed, semantically speaking, chronic, continuous residual intravascular hemolysis (or persistence of hemolysis seen in PNH patients carrying the R885H C5 polymorphism) (29) must not be defined as a breakthrough. PNH erythrocytes are exquisitely susceptible to complement-mediated lysis because they lack complement regulators, which are normally expressed on cell surface through a GPI anchor. Among all the GPI-linked proteins which are missing on PNH erythrocytes, there are the two main complement regulators CD55 (also named Decay Accelerating Factor, DAF) and CD59. As a result, both early complement activation (i.e., both assembly and decay of the C3 convertases C3bBb and C4b2a, regulated by CD55) (9–11) and terminal complement with its effector mechanism (i.e., assembly of the lytic C5b-C9 MAC, regulated by CD59) (12, 13) are uncontrolled, eventually leading to intravascular hemolysis (Figure 1) (1). Thus, the reappearance of intravascular hemolysis is mechanistically due to lack of effective complement inhibition; the event develops acutely because the PNH erythrocyte mass susceptible to complement activation is large (the proportion of affected erythrocytes increases during effective eculizumab treatment) (18–20). In the clinic, two distinct types of breakthrough hemolysis can be identified, which eventually imply different pathogenic mechanisms (Table 4) (44).

**Table 4:** Definition of pharmacokinetic and pharmacodynamic *breakthrough* hemolysis during eculizumab treatment for PNH.

	**Timing**	**Frequency**	**Concomitant conditions**	**Free C5**	**Eculizumab plasma level**	**Mechanism**	**Intervention**
Pharmacokinetic breakthrough	>7–10 days from previous dosing	Recurrent	Usually none^*^	Always >0.5–1 μg/mL	Inadequate	Residual free C5 available for steady-state (normal) C5 convertase activity	Decrease interval of dosing (10-12 days) or increase dose of eculizumab (1,200 mg)
Pharmacodynamic breakthrough	Any time	Sporadic	Infectious events (both bacterial and viral, such as common seasonal viruses) or any event leading to inflammation (i.e., surgery, possible comorbidities)	Usually ≤ 0.5–1 μg/mL (but it may occur with any free C5 plasma level)	Adequate	Massive complement activation leading to excess C5 convertase activity, which might displace C5 from eculizumab	None (treat the underlying cause triggering complement activation)

* *Events leading to pharmacodynamic breakthrough (i.e., triggers of complement activation) may eventually contribute also to pharmacokinetic breakthrough*.

The first (and better defined) example of breakthrough hemolysis was described in about 10–15% of PNH patients on eculizumab, as the frequent (and somehow regular) reappearance of hemolysis in the few hours/days before the next administration of eculizumab without any obvious trigger or complement activating conditions (i.e., LDH increases by 2–3 folds as compared to values assessed at day 7 from previous eculizumab dosing). In this case, impaired C5 blockade has been associated with low trough plasma levels of eculizumab demonstrated at 12–14 days from the previous dosing (42); thus, the term “***pharmacokinetic(PK) breakthrough***” has been designated for this condition (44, 45). Notably, the final confirmation that this is a PK phenomenon comes from the observation that changes to the treatment schedule (i.e., decreasing the interval dosing to 10–12 days, or increasing the dose to 1,200 mg) eventually result in sustained C5 blockade, with evident clinical benefit (21, 42, 46).

The second type of breakthrough hemolysis during anti-complement treatment in PNH is rather more unpredictable, since it may occur at any time (with respect to last infusion of eculizumab) and it tends to be sporadic and not recurrent as the PK breakthrough. In most cases, it is associated with infectious episodes or other clinical conditions that trigger complement activation in addition to the basal, low-grade, steady-state activation deriving from C3 tick-over (47–50). For this condition, we have used the term “***pharmacodynamic(PD) breakthrough***” (31, 44, 45) since it appears to be caused by massive complement activation, exceeding the inhibitory ability of eculizumab that is independent of its plasma level. The most frequent causes triggering this massive complement activation are infectious events (both bacterial and viral, such as common seasonal viruses) or any event leading to inflammation (i.e., surgery, possible comorbidities). In the clinic, it is well-accepted that in this condition extra dosing of eculizumab is not appropriate, because these episodes tend to be self-limiting, and the temporary increase in plasma level of eculizumab is not necessarily effective, as the plasma drug levels are already high. Notably, these acute events mirror the well-known hemolytic paroxysms which gave the name to the disease in absence of treatment, explaining the concept that the degree of complement activation is fluctuating acutely during these “trigger” events and may became clinically significant even during anti-C5 treatment. Experimental observations that aid in the understanding of this phenomenon are available both in the recent and old scientific literature. Indeed, we have documented that eculizumab at the therapeutic dose does not result in complete inhibition of hemolysis in an *in vitro* model investigating hemolysis of PNH erythrocytes (51–53). Recently, we have further dissected this phenomenon, using different conditions of complement activation *in vitro*, in the presence of eculizumab: whereas with spontaneous complement activation (paralleling the spontaneous, low-grade complement activation in steady-state clinical conditions) residual hemolysis is minimal (i.e., corresponding to the clinical finding of LDH ranging between 0.75 and 1.5 fold ULN); after massive complement activation hemolysis remains >40%, even with excess concentrations of eculizumab (5–10 times the therapeutic plasma levels) (44, 51). Thus, in clinical circumstances triggering the complement cascade, excessive complement activation may override the C5 blockade delivered by eculizumab, eventually leading to hemolytic crises due to PD breakthrough. The actual mechanism by which massive complement activation overrides eculizumab requires a review of complement biology: C5 is the substrate of an enzymatic reaction catalyzed by the C5 convertase, which eventually enables MAC formation. Anti-C5 antibodies bind to C5 in its fluid phase, preventing its cleavage by the C5 convertase, either of the alternative or of the classical/mannose pathway (17). Thus, PK and PD of eculizumab depend not only on its target C5, but also on the degree of C5 convertase activity competing with eculizumab for free C5, which in turn varies based on the magnitude of complement activation (54). It is quite obvious that complement activation may increase the number of C5 convertases; but it is even more important that the affinity of C5 convertases for the substrate C5 may vary also, as membrane-bound convertases have a much higher affinity for C5 as compared with convertases in the fluid phase (55, 56). Moreover, this affinity is largely dependent on the density of surface-bound C3b: when this density increases (by 10–100 times, in presence of events triggering complement activation) the excess of C3 generates very high-affinity C5 convertases (57–59). These complexes with high C3b content, which are generated at high rate on PNH erythrocytes even in presence of C5 blockade (see below) (28, 44), may displace C5 from the complex eculizumab:C5 thus arming the MAC, irrespective of therapeutic plasma levels of eculizumab and of very low levels of free C5 (57–59). Indeed, this breakthrough may be referred also as “*breakthrough with minimal free C5 levels*”; however, while PK breakthrough is unequivocally marked by high free C5 levels, PD breakthrough may contribute to hemolysis even when free C5 levels are high (C5 cleavage by high-affinity C5 convertases is further increased in excess of substrate). This mechanism of endogenous regulation of C5 convertase activity has been demonstrated not only for the alternative pathway convertase C3bBbC3b, but also for the classical/lectin pathway one C4bC2aC3b (59–61). These mechanistic data have been recently reproduced in the context of therapeutic C5 inhibition (62); interestingly, at least *in vitro*, the association of two different C5 inhibitors (that are both only partially effective if used in monotherapy) appears to overcome this phenomenon (62). Taken together, all these data support the existence of a breakthrough hemolysis due to PD reasons (i.e., secondary to transient massive complement activation); however, the clinical relevance of this phenomenon as well as possible therapeutic strategies for its prevention remain to be delineated.

#### Therapeutic goals

Residual intravascular hemolysis may persist during eculizumab treatment, either as low-grade continuous hemolysis or as breakthrough hemolytic crisis due to PK or PD reasons, which may eventually impact hematological response (see Table 1). With the exception of recurrent PK severe breakthrough (namely requirement for transfusion), which obviously requires therapeutic intervention to improve hematological response (Table 2), the other conditions may be clinically mild, and the actual need to develop novel strategies specifically targeting these conditions is questionable. Thus, while the possible impact of all novel therapies on residual intravascular hemolysis has to be addressed, we have to acknowledge that residual intravascular hemolysis seems not to be the most pressing unmet clinical need during eculizumab treatment.

### C3-Mediated Extravascular Hemolysis

Both residual intravascular hemolysis due to suboptimal C5 blockade and inadequate compensatory erythropoiesis due to underlying bone marrow failure may contribute to persistent anemia in PNH patients on eculizumab (31). However, most patients exhibit reasonable control of intravascular hemolysis (LDH stably <1.5 times the ULN) and adequate reticulocytosis (largely >100,000/μL). In contrast, all patients share a novel phenomenon which is the opsonization of surviving PNH erythrocytes with C3 fragments, which are detectable by flow cytometry (28, 63). Based on this finding, together with the demonstration of reduced *in vivo* half-life of ^51^Cr-labeled erythrocytes (with increased hepatosplenic uptake of ^51^Cr), we have described C3-mediated extravascular hemolysis as a novel disease mechanism which limits hematological benefit in most PNH patients on eculizumab (28, 64–66). C3-mediated extravascular hemolysis in PNH patients on eculizumab (or any anti-C5 agent) is not a complication, but rather a mechanistic phenomenon related to complement biology. We have discussed that PNH erythrocytes lack both CD55 and CD59 from their surface, and thus they are unable to control both early complement activation (i.e., assembly and decay of C3 and C5 convertases) and effector mechanisms of the terminal complement pathway (i.e., MAC assembly). Irrespective of the hierarchical contribution of CD55 and CD59 (the latter appears to be the most important surface endogenous complement modulator, at least for lysis prevention) (67), therapeutic C5 blockade prevents only MAC assembly, without interfering with early steps of the complement cascade. Thus, while PNH erythrocytes are kept alive by eculizumab because their lysis is precluded, surface complement activation on affected cells continues (mostly due to the constitutively active C3 tick-over of the alternative pathway), with covalent binding of C3b to erythrocyte surface (via glycophorin A, for example) and further generation of C3 convertase, which in turn amplifies C3 activation and C3 surface deposition. Then, PNH erythrocytes are progressively opsonized with different C3 split fragments (initially C3b, which then is quickly processed to C3d) (52), and they can be specifically recognized by C3 receptors (e.g., complement receptor 3) (68) leading to entrapment by professional phagocytes in the liver and spleen (28, 31, 64, 68). Different groups have confirmed opsonization by C3 split fragments as a common event in PNH patients on eculizumab (Figure 1B) (28, 66, 69); but its clinical relevance is not universally acknowledged by all experts (42, 70). The extent of this chronic extravascular hemolysis is very heterogeneous among patients, and the actual hemoglobin level reflects also residual intravascular hemolysis as well as compensatory erythropoiesis (even patients with normal hemoglobin levels exhibit massive reticulocytosis with increased bilirubin) (31, 64, 71). The clinical impact of this chronic anemia on quality of life (e.g., possible differences in distinct hematological response categories) and long-term organ damage has not been systematically investigated, even if some possible complications have emerged, such as iron overload, especially in patients still requiring transfusions (72–74). Inherited polymorphisms of different genes associated with complement regulation may shape the individual susceptibility of PNH patients to C3-mediated extravascular hemolysis; we have already shown that PNH patients carrying the hypomorphic variant of the complement receptor 1 gene have a much lower chance in achieving a good hematological response during eculizumab treatment (75). Given the number of proteins involved in complement activation and regulation (e.g., complement factor H, complement factor H related proteins, complement factor B, complement factor I, membrane cofactor protein, C3, etc.) (76, 77), it is likely that other gene variants associated with better or worse hematological benefit (as well as with residual intravascular hemolysis) may be identified in the near future.

#### Therapeutic goals

To date, there is no treatment option for C3-mediated extravascular hemolysis. The chronic use of steroids has been discouraged because of inefficacy and unacceptable side effects (65); splenectomy has been reported as possibly effective to ameliorate this condition (78, 79), but it is not considered a standard treatment (70). Thus, C3-mediated extravascular hemolysis represents an unmet clinical need in PNH, and it is the most reasonable therapeutic goal for any new strategy of complement inhibition.

## The Role Of Bone Marrow Transplantation

Bone marrow transplantation (BMT) remains the only curative treatment for PNH (32, 80, 81), however its use is limited by treatment-related morbidity and mortality. As discussed above, BMT is a key treatment option in patients with AA/PNH syndrome; however, its role can be discussed even in patients with classic PNH. Indeed, the outcome of patients undergoing allogeneic BMT for classic, purely hemolytic, PNH is excellent, with a long-term survival of 80–90% (32). BMT remains the best treatment option for hemolytic PNH for patients who have no access to eculizumab treatment, which is the case for many developing countries. Indeed, for emerging markets the very high price of eculizumab (82) represents a major limitation to its use, even with approval from regulatory authorities (which does not necessarily imply allocation of financial resources and reimbursement). Since the cost of BMT can be equivalent to about 3–4 months of eculizumab treatment, BMT may be not only clinically appropriate, but even cost-effective. In addition, BMT might be considered even where eculizumab is fully available, in case of lack of hematological response to the treatment; however, no response (see Table 1) is rare, and even in case of minor hematological benefit eculizumab appears to retain obvious clinical benefits, with major impact on long-term survival (23, 24). Thus, BMT is not recommended for the majority of hemolytic PNH patients with unsatisfactory hematological response; and for this condition, novel strategies of complement inhibition represent an intriguing alternative to BMT.

## THE Second Generation Of Anti-Complement Agents For PNH

The clinical development of eculizumab for PNH, and then also for other diseases, has been a unique experience in terms of both scientific and financial success. This growing interest in the field of complement therapeutics has generated several preclinical and clinical programs for the development of novel anti-complement agents (Table 5 and Figure 1). We have already reviewed quite recently most of these programs (45, 126); here we focus on the possible therapies whose development appears more advanced, or more exciting for their possible breakthrough results. Indeed, our discussion is biased by our commitment to address the major unmet clinical needs in PNH, as described in the first part of this manuscript. Therapeutic agents interfering with complement activity may be grouped based on different factors; for this review, the most useful classification is based on their targets in the complement cascade. Two major classes of complement inhibitors may be identified: (i) inhibitors of the terminal complement pathway targeting C5 (even if agents targeting downstream complement components such as C6 have been announced); (ii) inhibitors of early phases of the complement cascade targeting either the key event of the cascade (C3 cleavage), or pathway-specific initiating events (for PNH, they include proteins of the alternative pathway such as complement factor D, factor B and properdin); all together, these agents can be classified as proximal complement inhibitors.

**Table 5:** Complement inhibitors in clinical development for PNH.

**Class**	**Agent**	**Target**	**Clinical trial ID**	**Design**	**Patient population**	**Study treatment**	**Results**
Terminal inhibitors	ALXN1210	C5	N.A.	Phase I, randomized vs. placebo	Healthy volunteers	SAD, IV infusions	Yes
			NCT02598583 (83)	Phase I/II, open-label	Untreated PNH	Intra-patient DE by IV infusions	Yes (84)
			NCT02605993 (85)	Phase I/II, open-label	Untreated PNH	MAD; IV infusions	
			NCT02946463 (86)	Phase III, randomized vs. Ecu	Untreated PNH	IV infusions (every 8 weeks)	Yes (87)
			NCT03056040 (88)	Phase III, randomized vs. Ecu	Stable responders PNH	IV infusions (every 8 weeks)	Yes (89)
	SKY59	C5	NCT03157635 (90)	Phase I/II, multi-part study	Healthy volunteers	SAD, IV infusions	Yes (91)
					Untreated PNH	Intra-patient DE by IV infusions, followed by SC injections	Yes (92)
					Stable responders PNH		
	LFG316	C5	NCT02534909 (93)	Phase II, open-label	Untreated PNH	IV infusions	Pending
	REGN3918	C5	NCT03115996 (94)	Phase I	Healthy volunteers	IV and SC infusions	Yes (95)
	ABP959	C5	EudraCT 2017-001418-27 (96)	Phase III, randomized vs. Ecu	Stable responders PNH	IV infusions	Ongoing
	RA101495	C5	N.A.	Phase I, SAD and MD	Healthy volunteers	Daily, SC injections	Yes (97, 98)
			NCT03078582 (99)	Phase II, open label, fixed dose	Untreated PNH	Daily, SC injections	Yes (100)
					Poor responders PNH		
			NCT03030183 (101)	Phase II, open label, fixed dose	Poor responders PNH	Daily, SC injections	Ongoing
			NCT03225287 (102)	Phase II, open-label, extension	PNH exposed to RA101495	Daily, SC injections	Ongoing
	Coversin	C5	N.A.	Phase I, SAD and MD	Healthy volunteers	SC injections	Yes (103)
			NCT02591862 (104)	Phase II, open-label	Poor responder PNH	SC injections; intra-patient DE	Pending
			EudraCT 2016-002067-33 (105)	Phase II, open-label, fixed dose	Untreated PNH	SC injections	Yes (106)
			EudraCT 2016-004129-18 (107)	Phase II, open-label, extension	PNH exposed to coversin	SC injections	Ongoing
	ALNCC5	C5	NCT02352493 (108)	Phase I/II, randomized vs. Ecu, SAD and MAD	Healthy volunteers	SC injection (ALNCC5 or placebo)	Yes (109)
					Untreated PNH	SC injections (ALNCC5 only)	Yes (110)
			EudraCT 2016-002943-40 (111)	Phase II, open-label	Poor responder PNH	SC injections	Pending
Proximal inhibitors	TT30	CAP	NCT01335165 (112)	Phase I, SAD	Untreated PNH	SC injections and IV infusions	Yes (113)
	AMY-101	C3	NCT03316521 (114)	Phase I, SAD and MD	Healthy volunteers	SC and IV infusions	Pending
	APL-2	C3	N.A.	Phase I, SAD and MD	Healthy volunteers	SC and IV infusions	Yes (115)
			NCT02264639 (116)	Phase Ib, open label, MAD, POC	Poor responders PNH	Daily, SC infusions	Yes (117)
			NCT02588833 (118)	Phase Ib, open label, MAD, POC	Untreated PNH	Daily, SC infusions	
			NCT03531255 (119)	Phase III, open label, extension	PNH exposed to APL-2	Daily, SC infusions	
			NCT03500549 (120)	Phase III, randomized vs. ecu	Poor responders PNH	SC infusions, BIW	Ongoing
	ACH-4471	FD	N.A.	Phase I, SAD	Healthy volunteers	Orally, QD and BID	Yes (121)
			NCT03053102 (122)	Phase II, open label, MD, POC	Untreated PNH	Orally, TID	Pending
			NCT03181633 (123)	Phase II, open-label, extension	PNH exposed to ACH-4471	Orally, TID	Ongoing
			NCT03472885 (124)	Phase II, open label, MD, POC	Poor responders PNH	Orally, TID	Ongoing
	LNP023	FB	NCT03439839 (125)	Phase II, open label	Poor responders PNH	Orally, BID	Ongoing

*N.A, not available; Ecu, eculizumab; SAD, single ascending dose; MAD, multiple ascending doses; MD, multiple doses; POC, proof-of-concept; DE, dose escalation; SC, subcutaneous; IV, intravenous; QOD, quaque die (once a day); BID, bis in die (twice a day); TID, ter in die (thrice a day); BIW, bis in week (twice a week); PK, pharmacokinetics; PD, pharmacodynamics; LDH, lactate dehydrogenase*.

### Novel Inhibitors of the Terminal Complement

There are at least seven novel anti-C5 agents (in addition to biosimilars of eculizumab, which have been announced as well), which have entered clinical development for PNH; most of them are monoclonal antibodies like eculizumab, but the list includes also small peptide inhibitors and small interfering RNA (siRNA). All these agents aim to reproduce the excellent data achieved with eculizumab, trying to address some other clinical needs mostly concerning patient (dis)comfort: indeed, current eculizumab treatment requires intravenous (IV) infusions given every 14 days indefinitely. These agents have been designed trying to increase the interval between administrations, and/or switching from an IV dosing to a subcutaneous (SC) or even oral one.

#### ALXN1210 (Ravulizumab)

ALXN1210 (also known with the brand name of ravulizumab, Ultomiris®) is the first of the second-generation therapeutic complement inhibitors, as well as the one with the most advanced clinical program. ALXN1210 is another anti-C5 mAb which was generated through specific amino acid modifications of eculizumab aiming to improve its PK profile (127). The half-life of eculizumab is largely influenced by non-specific pinocytosis by endothelial cells and trafficking into the lysosome compartment, where it can be efficiently degraded (especially if bound to its target C5) (127). A potential strategy to reduce this degradation and to favor recycling to the vascular compartment may exploit a more efficient dissociation of the mAb:C5 immune complex in the low pH lysosome compartment, together with a higher affinity for the neonatal Fc receptor (FcRn), which is responsible for specific recycling to the extracellular, vascular compartment (127). ALXN1210 was designed to exploit targeted reengineering of eculizumab: two histidine switches were included in the complementary determining regions (CDRs) to promote more efficient pH-dependent dissociation of the mAb:C5 complex, and two additional amino acides changes were included in the Fc region to increase the affinity for the FcRn (127). Based on preclinical data, both these goals have been achieved. ALXN1210 exhibited a reduced target-dependent drug disposition (TDDM) and a longer half-life as compared to its parental molecule eculizumab, becoming an attractive long-acting anti-C5 mAb to be used in the clinic (127). Two phase Ib/II multicenter studies were conducted to investigate safety and preliminary efficacy of different IV doses of ALXN1210 in untreated PNH patients (84). In the first study (NCT02598583, study 103), 13 PNH patients received the drug every 4 weeks at the maintenance dose of either 900 (same as eculizumab) or 1800 mg (83); in the second study (NCT02605993, study 201), 26 PNH patients were treated with the maintenance dose of 1,000 mg every 4 weeks, 1,600 mg every 6 weeks, 2,400 mg every 8 weeks, or 5,400 every 12 weeks (85). Without focusing on details, rapid and sustained reduction in LDH (which was the primary endpoint) was achieved in all cohorts, without substantial difference in percentage change from the baseline (84). However, the percentage of patients achieving normal or near-normal LDH (<1.5 times of the ULN) was higher in those with the higher trough exposure to ALXN1210 (i.e., 1,800 mg every 4 weeks) (84). The safety profile of ALXN1210 was overlapping to that established for eculizumab, with no deaths, and no adverse events (either serious or non-serious) leading to drug discontinuation; however, two cases of sepsis by *N. Meningiditis* were observed (both patients completely recovered after ceftriaxone treatment, and continued ALXN1210 therapy) (84). These results led to the design of two large phase III, open-label, randomized trials where the maintenance dose of ALXN1210 was 3,300 mg every 8 weeks (with some dose adjustment based on patient weight: 3,000 mg for those <60 kg, 3,600 for those >100 kg); the data of these studies have become available recently (87, 89).

The study 301 (NCT02946463) tested for non-inferiority of ALXN1210 as compared with eculizumab in treatment-naïve hemolytic (LDH >1.5 times of the ULN) PNH patients (86). A total of 246 patients were randomized 1:1 to receive either ravulizumab (a single loading dose of 2,700 ± 300 mg for under- or overweight patients was followed by maintenance dose starting after 2 weeks) or eculizumab for 6 months; the co-primary endpoints were transfusion independence and LDH normalization (86). ALXN1210 was non-inferior to eculizumab for both the primary endpoints (transfusion independence, 73.6 vs. 66.1%; LDH normalization, 53.6 vs. 49.4%, with *P* for non-inferiority <0.001) as well as for additional efficacy secondary endpoints including percent reduction of LDH, stabilized hemoglobin, breakthrough intravascular hemolysis, and quality of life measures (86). No meningococcal infection was observed, with excellent safety and tolerability for the 8-week interval regimen (9). The twin study 302 (NCT03056040) was conceived as a switch-study for PNH patients already on eculizumab, assessing the non-inferiority of ALXN1210 vs. eculizumab in PNH patients in clinically stable conditions on standard-of-care eculizumab treatment (i.e., 900 mg every 14 ± 2 days) (88). A total of 195 patients were randomized 1:1 to switch to ALXN1210 or continue on eculizumab; the primary endpoint was percentage change in LDH (88). Considering the 191 patients who have completed the 6 month treatment, ALXN1210 was non-inferior to eculizumab: percentage change in LDH compared to baseline was minimal in both arms (−0.82% for ALXN1210 vs. +8.39% for eculizumab), with non-inferiority shown also for all efficacy secondary endpoints (hemoglobin stabilization, breakthrough intravascular hemolysis, and quality of life measures) (88). The safety profile was excellent, with no cases of meningococcal infection recorded in this trial; interestingly, the most common AE was headache (26.8% with ALXN1210 vs. 17.3% with eculizumab). Headache is commonly observed after starting of eculizumab, as a result of inhibition of intravascular hemolysis with reduced release of free hemoglobin and sudden increase of circulating nitric oxide (128); the observation that headache may be seen also upon switching from eculizumab to ALXN1210 may suggest that in some patients a deeper control of intravascular hemolysis has been achieved (88). Based on these data both in untreated PNH patients and as switch-therapy from eculizumab, ALXN1210 has received marketing authorization by the FDA (Ultomiris®, Alexion Pharmaceuticals, New Haven, CT, USA) and approval by EMA is expected soon. Furthermore, an additional trial investigating SC dosing of ALXN1210 has been announced (129).

#### SKY59/RO711268/Crovalimab

SKY59 (also known as RO711268 or Crovalimab, in development by Roche) is another long-acting anti-C5 mAb, which exploits a pH-dependent binding to the target C5, eventually accounting for profound mAb recycling (130, 131). This mAb has been generated with the same goal of using histidine residues to achieve a pH-dependent binding to the antigen, eventually favoring the dissociation of the immune complex in the low pH lysosome compartment through changes in the surface charge of the mAb (131). This increased dissociation promotes the degradation of the C5 released into the lysosomes (thereby preventing C5 accumulation commonly seen with eculizumab treatment), and the recycling of the mAb to the plasma through its recognition by the FcRn (130, 131). The engineered SKY59 mAb generated with this technology exhibits a much longer half-life *in vivo* in cynomolgus monkeys, accounting for a sustained C5 blockade even after SC administrations (130, 131). Since SKY59 binds C5 epitopes different from eculizumab, SKY59 has been shown to efficiently block even the R885H polymorphic C5variant, at least *in vitro* (130). The clinical development of SKY59 in PNH has been pursued through a complex phase I/II study (NCT03157635) consisting of three sequential parts and an open-label extension (90). In part 1, SKY59 was investigated in healthy volunteers, whereas parts 2 and 3 enrolled untreated and eculizumab-treated PNH patients, respectively; in all three parts safety, tolerability, PK/PD and efficacy of SKY50 was evaluated (90). Data on the first two parts of the study have been recently presented (91, 92). Part 1 was a randomized, placebo-controlled, single ascending dose study to evaluate safety, tolerability, PK/PD of SKY59 in healthy subjects; three dose cohorts of five subjects were investigated: 75 and 125 mg given IV, and 100 mg given SC (91) Single ascending doses of SKY59 were well-tolerated, without severe or serious adverse events; exposure was dose proportional in the two IV dose levels, with a terminal half-life of about 25 days (91). After the SC dosing, bioavailability was estimated around 90%, with peak plasma levels being achieved after 7 days (91). Dose-dependent inhibition of the terminal complement activity was observed, with transient complete inhibition demonstrated in 2 out of 3 subjects receiving 125 mg IV (91). Part 2 of the study consisted in an intra-patient dose escalation, with SKY59 given at the doses of 375, 500 and 1,000 mg on days 1, 8 and 22, followed by weekly SC maintenance doses of 170 mg from day 36 (92). In the 10 eculizumab-naive PNH patients enrolled in part 2, half-life of SKY59 was confirmed as 25 days; complete terminal complement inhibition was observed after all IV and SC dosing (92). All these untreated PNH patients (one carrying the R885H C5 polymorphism) achieved a marked reduction of intravascular hemolysis, as demonstrated by a median LDH reduction of 79%; after 6 weeks of treatment, LDH remained in a range between 0.8 and 1.7 times of the ULN (92). In part 3 of the study PNH patients on eculizumab were switched to SKY59 with an IV loading dose of 1,000 mg (given 2 weeks after the last administration of eculizumab), followed by a randomization for maintenance SC SKY59 at 3 different schedules: 170 mg weekly, 340 mg every 2 weeks and 680 mg every 4 weeks (92). Sixteen eculizumab-treated PNH patients were enrolled; during SKY59 treatment, they all maintained LDH levels similar to those recorded during eculizumab therapy, except patients carrying the R885H C5 variant, who as expected achieved a major reduction (92). Two patients in part 3 developed drug-target-drug complex (DTDC) mediated reactions, with vasculitis-like symptoms similar to serum sickness; they appeared at 9 and 10 days from loading dose of SKY59 due to SKY59-C5-eculizumab complexes generated during the (transient) concomitant presence of the 2 anti-C5 mAbs during the switching period. These adverse events required topical treatment with steroids and resolved within 3 weeks without any sequelae, with no SKY59 discontinuation (92). Taken together, results from parts 2 and 3 demonstrated that SKY59 is an effective C5 inhibitor with excellent bioavailability after SC low-volume dosing (given weekly or even with longer intervals)(92). Hemoglobin levels rose by at least 1 g/dL in untreated patients, and remained stable in patients switching from eculizumab (92). A few episodes of breakthrough intravascular hemolysis were observed, mostly associated with concomitant events triggering complement activation (i.e., PD breakthrough, as confirmed by data on free C5, which remained low in all treated patients). Quite interestingly, in contrast to free C5, plasma levels of total C5 exhibited significant changes during treatment: whereas in untreated patients there was an increase from 140 μg/mL (73.6–184 μg/mL) to 215 μg/mL (109–331 μg/mL), in patients switching from eculizumab a reduction from 295 μg/mL (205–354 μg/mL) to 228 μg/mL (184–305 μg/mL) was observed (92). These findings, although preliminary, appear to demonstrate the specific C5 disposing activity of SKY59 in comparison to eculizumab (92, 131); however it has to be acknowledged that in this study the total C5 levels seen on eculizumab appeared to be quite high compared to what was observed in other studies (132).

#### LFG316

LFG316 is another anti-C5 mAb in development by Novartis; this agent is currently under investigation in PNH patients within a proof-of-concept phase II study enrolling untreated PNH patients (93). The study exploits LDH change as primary endpoint, looking for preliminary efficacy of LFG316; since Japanese centers are actively involved, this study aims to address the unmet clinical need for PNH patients intrinsically resistant to eculizumab due to the R885H C5 polymorphism (29). Further details on the study (e.g., PK of LFG316 after systemic injection; this agent has been initially developed for local use in age-related macular degeneration) as well as preliminary results are not yet available.

#### REGN3918

REGN3918 is an anti-C5 mAb in development by Regeneron, which binds both wild-type and R885H variant of human C5. In a phase I study in healthy volunteers REGN3918 was well-tolerated and resulted in dose-dependent inhibition of the terminal complement pathway, measured as hemolytic activity (CH50) (95). This agent exhibits a favorable PK profile, since it is bioavailable even after SC administration; a single IV loading dose followed by weekly SC dosing resulted in sustained inhibition of C5 activity (95). Further trials in PNH have been announced.

#### Biosimilars of Eculizumab

In addition to novel anti-C5 mAbs, biosimilars of eculizumab have also been described. For instance, ABP959 is a biosimilar of eculizumab developed by Amgen; this agent is now under investigation in a large Phase III trial (96). Another biosimilar of eculizumab (SB12) has been announced by Samsung Bioepis^1^.

#### RA101495

RA101495 is the lead compound of a new class of small synthetic, macrocyclic peptides developed by Rapharma to inhibit C5 (133); preclinical data have demonstrated efficacy in preventing PNH hemolysis *in vitro* (134). RA101495 was safe and well-tolerated in healthy volunteers after single SC administrations (97); multiple daily SC administrations were confirmed safe, and resulted in complete (>95%) and sustained inhibition of the terminal complement pathway (98). The first study investigating RA101495 in PNH (NCT03078582) enrolled both untreated and eculizumab-treated PNH with evidence of hemolysis (LDH >2 times of the ULN) (99); patients received RA101495 as SC injections, with a loading dose of 0.3 mg/kg followed by 0.1 mg/kg daily (possibly escalated up to 0.3 mg/kg) (99). The study enrolled 10 untreated and 16 eculizumab-treated PNH patients; the latter received RA101495 as a switch therapy from eculizumab (100). All the 10 untreated patients achieved a major reduction in LDH, with median LDH stabilized around 1.5–2 times of the ULN (thus, residual intravascular hemolysis remained evident) (100). In PNH patients switching from eculizumab, LDH response was observed as well, even if transfusion-dependence on eculizumab was associated with subsequent breakthrough hemolysis on RA101495 (100). The authors reported that 16 of the 21 patients completing the 12-week study have continued the treatment with RA101495 within an extension study (102). In addition to this trial, a second study on PNH (NCT03030183) investigated the same treatment regimen as add-on treatment in PNH patients with inadequate response to eculizumab (101). According to the latest update, three patients have been enrolled (100); results from this latter study have not been reported yet. In the meantime, additional anti-C5 macrocyclic peptides have been developed with excellent oral bioavailability (135); preclinical data suggest that exposure levels needed for therapeutic efficacy in humans may be reached (136).

#### Coversin

Coversin is another recombinantly expressed inhibitor of C5, which originates from the tick *Ornithodoros moubata*; this 16 kDa protein binds C5, thereby preventing its cleavage by all C5 convertases (137). Its potential efficacy in PNH is supported by *in vitro* data showing that coversin may prevent lysis of PNH erythrocytes (103), even in samples from patients carrying the R885H C5 polymorphism (138). Coversin has shown excellent bioavailability after SC administration, without safety concern (103); thus, a clinical program in PNH was started. The first proof-of-concept study was successfully conducted in PNH patients resistant to eculizumab due to the C5 polymorphism (NCT02591862) (104). Then a Phase II single arm, open label trial was conducted in previously untreated PNH patients; this study investigated coversin given SC with a dose-adaptation based on adequate control of hemolysis (105). Indeed, the protocol included an initial loading regimen (single loading dose of 60 mg, followed by 1–3 doses at 30 mg every 12 h), followed by a bi-daily regimen with 15 or 22.5 mg; then from day 29 patients switched to a daily regimen at the dose of 30 or 45 mg (106). In case of suboptimal inhibition patients had the option to increase the daily dose, or split it into bi-daily dosing; after the 3-month treatment, an extension study was made available for all patients willing to continue their coversin treatment (107). Five patients were treated according to the planned schedule, but control of hemolysis during the first month of treatment appeared suboptimal (106); thus the protocol was amended to give 22.5 mg bi-daily to all patients starting from 12 h after the ablative regimen. In general, coversin was very well-tolerated, with mild injection site reaction as the only adverse event (they were self-limiting, and reduced in severity over time); even if anti-drug antibodies were rarely seen, no neutralizing antibodies were detected (106). The primary endpoint of reducing LDH below 1.8 times ULN was achieved (as median value of the full cohort; taken individually, 5 of the 8 patients achieved an LDH value <1.8 times of the ULN); nevertheless, LDH ranged around 1.2–1.8 times of the ULN, without normalization (106). Of the eight patients enrolled, one patient withdrew due to comorbidity unrelated with coversin treatment, and seven decided to continue the treatment within the extension study (107); six are currently receiving a daily dose of 45 mg, and one of 30 mg (106). Self-administration was achieved in all patients, without any need of hospitalization to deliver the treatment.

#### ALN-CC5

In addition to mAbs and small peptide molecules, another strategy of C5 inhibition was developed aiming to interfere with endogenous C5 production by RNA interference. The first-in-class agent for this strategy is ALN-CC5, a si-RNA duplex specific for C5, that had been shown highly effective in silencing liver C5 production in animal models (139). The clinical program of ALN-CC5 started with a phase I/II trial (NCT02352493) enrolling both healthy volunteers and untreated PNH patients (108). In the 32 healthy subjects enrolled, ALN-CC5 was found to be safe and very effective, leading to >99% reduction of C5 plasma levels (109); this was associated with a profound inhibition (>95%) of serum complement activity (109). Thus, investigation in PNH was started with a high degree of enthusiasm; within the same trial (108) six PNH patients were treated with ALN-CC5 at the weekly SC dose of 200 or 400 mg (110). Among these patients, three were treatment-naïve (and thus were treated in monotherapy), whereas three received ALN-CC5 as add-on treatment to eculizumab (110). The treatment was safe, since no adverse event required treatment discontinuation; results were different in the two patient populations, irrespective of the fact that in all patients C5 production was inhibited by >98% (110). In previously untreated PNH patients C5 knockdown was established slowly, and therapeutic inhibition required about 2 months to appear (110); moreover, inhibition of intravascular hemolysis remained partial, since LDH reduction (37 and 50%) was observed only in 2 of 3 patients (both starting with LDH >5 times ULN), and all patients continued to show LDH stably >1.5 times of the ULN (110).These findings are consistent with *in vitro* data showing that, using C5-depleted sera, complement-mediated activity in PNH erythrocytes is fully restored with small amount of recombinant C5 as low as 0.9 μg/mL (about 1% of normal C5 plasma level) (62, 140). These patients were rescued by the addition of eculizumab at very low dose (600 mg every 4 weeks), supporting the concept that a combined treatment may be more effective. On the other hand, in the study three patients started ALN-CC5 as add-on treatment, for inadequate response to eculizumab; in these patients, combined treatment resulted in LDH normalization (110). This combined effect was seen also in one patient with chronic PK breakthrough, who retained LDH normalization even after reduction of eculizumab to the standard regimen of 900 mg every 2 weeks (110). All together, these data demonstrate that ALN-CC5 monotherapy may result in suboptimal control of intravascular hemolysis in PNH; on the other hand, once used in combination with eculizumab, ALN-CC5 allows for more effective C5 inhibition, which has not been seen so far with anti-C5 mAb. However, data about long-term duration of RNA-based C5 inhibition, as well as on efficacy of re-treatment, are lacking. The clinical investigation of this combined treatment with eculizumab and ALN-CC5 is currently ongoing in a trial enrolling poor responders to eculizmab, aiming to improve their clinical response (111).

### Inhibitors of the Proximal Complement

The development of proximal complement inhibitors has not been as intensely investigated as the search for novel anti-C5 agents (at least so far); now, there are only five clinical programs, which have been publicly disclosed, but only four remain active. Before listing and discussing them in detail, it is important to summarize how and why the idea of interfering with the proximal steps of the complement cascade became of interest in PNH. As discussed above, the critical understanding of therapeutic complement inhibition *in vivo* with eculizumab revealed that C3-mediated extravascular hemolysis may limit the hematological benefit of anti-C5 treatment (28, 64, 66). As the initial reporters of this event in PNH, we felt the need to address this problem therapeutically by hypothesizing that blockade of the complement cascade upstream of C5 may represent a promising strategy for treating C3-mediated extravascular hemolysis emerging in PNH during eculizumab treatment (141). Indeed, the field of complement provided several options as alternative targets in the complement cascade (142–144). Our work and that from others provided experimental work supporting the hypothesis that different proximal complement inhibitors may efficiently prevent C3-mediated extravascular hemolysis in PNH, likely also inhibiting concomitantly intravascular hemolysis (Figure 1C) (52, 53, 145, 146). The first proximal complement inhibitor to enter clinical development was TT30, a 65 kDa engineered protein which fused the functional domain of complement factor H (FH) with the iC3b/C3dg-binding domain of complement receptor 2 (147). This molecule was designed with the aim of delivering the inhibitory effect of FH at the level of complement activation (i.e., for PNH, the surface of erythrocytes binding C3b); our preclinical work confirmed that *in vitro* PNH erythrocytes were protected against both MAC-mediated lysis and C3 opsonization (52). In 2011, a phase I study (NCT01335165) was conducted to investigate tolerability, PK, PD, and immunogenicity of TT30 given as single IV infusion or SC injection in untreated PNH patients (112). Unfortunately, the results of this study have been published only in abstract form; TT30 was safe and well-tolerated, with no safety concern and no evidence of immunogenicity (113). PK and PD data demonstrated that pharmacological levels of TT30 may be achieved and are associated with inhibition of complement activity (including terminal complement pathway) (113). However, even if biological activity was seen as transient decrease of LDH (after single doses), the program was halted due to the extremely short half-life of the compound (113).

Currently the field of proximal complement inhibitors include broad inhibitors of C3 (with the two compstatin analogs AMY-101 and APL-2) and selective inhibitors of the alternative pathway targeting either complement factor D (FD) and complement factor B (FB).

#### AMY-101

AMY-101 is an analog of a 13-residue disulfide-bridged peptide named compstatin, discovered in the 90's by Prof. J. Lambris using a phage-displayed random peptide library (148). Compstatin binds to human and non-human primate native C3 and to its active fragment C3b, preventing the convertase activity of compstatin-bound C3bBb, and preventing also the cleavage of compstatin-bound C3 into C3b by pre-formed C3 convertases (148, 149). Compstatin and its analogs are broad inhibitors of C3, which completely disable the activation of the complement cascade along all the activating pathways, including the amplification loop (150). AMY-101 (previously known as Cp40) is the most recent generation analog of compstatin, which harbors increased affinity and potency and better PK features (151). The effect of AMY-101 in PNH has been initially investigated *in vitro*, where it is able to fully prevent C3 deposition on PNH erythrocytes and they are also protected against MAC-mediated lysis (53). Thus, this analog was identified as the best candidate for clinical development by Amyndas Pharmaceuticals. A single and multiple ascending dose phase I study to investigate safety, PK and PD of AMY-101 in healthy volunteers has been completed (NCT03316521) (114). According to company's announcement, AMY-101 was safe, well-tolerated, and exhibited a PK/PD profile which can support a therapeutic schedule of efficient complement C3 inhibition, via subcutaneous administration every 48 h (152). Phase II trials investigating the efficacy of AMY-101 as monotherapy in both untreated and eculizumab-treated (poor responders) PNH patients have been announced (153).

#### APL-2

APL-2 is another compstatin analog which utilizes a first-generation version of compstatin (151), modified through a pegylation aiming to increase its half-life *in vivo*. Two separate double-blinded, placebo-controlled, phase I studies investigated safety, tolerability, PK, and PD of single- and multiple ascending doses of APL-2 in healthy volunteers (115). In total, 24 subjects received single doses (ranging from 45 to 1,440 mg, SC) and 16 subjects received multiple daily SC doses (ranging from 30 to 270 mg) (115). No serious or severe adverse event was reported, nor events leading to study drug discontinuation (115) The exposure to APL-2 increased linearly with increasing doses, and steady-state plasma level were achieved after 28 days of daily dosing (115). PD was measured by *ex vivo* complement functional assay: complement inhibition was demonstrated with single doses of 1,440 mg, and with multiple doses of 180 and 270 mg (115). APL-2 was then investigated both in untreated and eculizumab-treated PNH patients (116, 118). The PADDOCK study (NCT02588833) investigated APL-2 as monotherapy in untreated PNH patients with meaningful intravascular hemolysis (defined as LDH >2 times ULN and Hb <10.5 g/dL) (118). APL-2 was given SC as daily infusions (to limit skin irritation), at doses of 180 (cohort 1) or 270 mg (cohort 2); three patients were enrolled in cohort 1 and 20 in cohort 2 (117). Reduction of LDH was observed in all patients, with 95% of patients achieving LDH normalization by day 29 of treatment; then, LDH remained in the normal range at all time points investigated (117). Similarly, hemoglobin was raised during treatment, increasing from median 8.0 g/dL at baseline to median 10.8 g/dL at day 29, and sometimes higher to a median of 12.2 g/dL when APL-2 treatment was continued (day 85) (117). Only a few patients (*n* = 4) required red blood cell transfusions while on APL-2 (2 prior to APL-2 steady-state, and 1 for concomitant AA), although 17 patients were transfusion dependent at enrollment (117). Other evidence of adequate control of both intravascular and C3-mediated extravascular hemolysis was the normalization of bilirubin, the reduction of absolute reticulocyte counts (which after an initial drop raised again just above the ULN), and even more the proportion of PNH erythrocytes, which started at 32% at baseline and progressively increased to 67% and up to 80% at day 29 and 85, respectively (117). These data demonstrate that systemic inhibition of C3 with APL-2 controls both intravascular and extravascular hemolysis in PNH, eventually leading to substantial hematological benefit. Another study is ongoing in PNH patients with inadequate response (defined as Hb level <10 g/L and/or the need for red blood cell transfusion) to eculizumab (PHAROAH study; NCT02264639); in this phase Ib trial APL-2 is investigated as add-on therapy on top of eculizumab (116). Final data from this trial are not yet available, despite the company announced that in six poor responders APL-2 treatment resulted in mild increase in hemoglobin and reduction of transfusion burden, with concomitant normalization of LDH (154). Globally, more than 5,000 SC doses of APL-2 (of 270 mg or higher) have been administered to PNH patients within these trials, for a systemic exposure >700 patient weeks. So far, no safety concern has emerged, and even the feared higher risk of infectious complications compared with terminal complement inhibition seems controlled (possibly because of the risk-mitigation strategy exploited by extended vaccination schedules and pharmacological antibiotic prophylaxis). Based on these data, Apellis has launched a large phase III trial in PNH patients with suboptimal hematological response to eculizumab, defined as Hb level <10.5 g/dL; in this study, after a short period of concomitant treatment (to reach APL-2 steady-state), patients will be randomized to continue either eculizumab or APL-2 monotherapy (120).

#### ACH-4471

ACH-4471 is small oral FD inhibitor developed by Achillion which showed inhibitory activity of hemolysis in PNH *in vitro* (155); it was selected among many candidate agents for its better PK profile (156). A first-in-human study was conducted in healthy volunteers as single ascending dose and 14-day multiple ascending doses; no safety issue emerged (121). Therapeutic doses were used in the range of 200–600 mg, which resulted in significant inhibition of the alternative pathway; bi- or tri-daily administration were anticipated to sustain pharmacological levels of the drug (121). The first study in PNH was a phase II trial enrolling untreated patients with clinically meaningful intravascular hemolysis (122). ACH-4471 was used as monotherapy; the primary endpoint of the study was change in LDH level, whereas hemoglobin level and C3 deposition were included as secondary endpoints (122). This study has completed its recruitment (planned for 10 patients) and results are expected before summer 2019; in the meantime, all enrolled patients are continuing ACH-4471 treatment within an extension study (123). In parallel, a second study was started in PNH patients with poor response to eculizumab (defined as the need of red blood cell transfusion); here ACH-4471 was given as add-on treatment on top of standard eculizumab at three different doses (100, 150, or 200 mg thrice a day) (124). In agreement with the actual reasons which led to the clinical development of proximal complement inhibitors in PNH, the primary endpoint of this trial is the change in hemoglobin levels (124).

#### LNP023

LNP023 is a small FB inhibitor that, together with small FD-inhibitors, constitutes Novartis' pipeline of potent and orally bioavailable selective inhibitors of the alternative pathway. Similarly to anti-FD agents (146), LNP023 prevents lysis and C3 opsonization of PNH erythrocytes *in vitro* (157). LNP023 is currently under investigation in a phase II trial as add-on therapy in patients with poor response to eculizumab, defined as LDH persistently >1.5 times of the ULN (125). This study aims to investigate safety, PK, PD and efficacy of LNP023 used at the fixed dose orally; while the primary endpoints has been set on better control of intravascular hemolysis (based on LDH values), C3 deposition and hemoglobin changes are included as secondary endpoints (125). The estimated enrollment for this study is 15 PNH patients (125); results are expected in summer 2019.

## Future Anti-Complement Treatment For PNH: Goals, Hopes And Guesses

These are exciting days for the field of PNH, and possibly in a few years new anti-complement therapies will change the standard care of this disease and possibly others. As new data are generated, we will better understand the benefits of complement cascade modulation, and what we should aim to do in the near future. Indeed, a couple of lessons are clear even at this stage of investigations.

### Hematological Response and Clinical Benefit During Eculizumab Treatment

Eculizumab treatment largely improves survival in PNH patients (23, 24); apparently, this effect is retained irrespective of the different hematological benefits achieved by individual patients. Thus, while a better hematological response is a worthy therapeutic goal, its possible impact on long-term survival cannot be anticipated, and (if any) may require very long follow up to be demonstrated. We emphasize that the categories of hematological response introduced in this manuscript are useful to identify patients who may benefit from novel strategies of anti-complement treatment, but have not been developed to identify patients who may discontinue current eculizumab treatment. While in these circumstances it seems worth looking for a better strategy to improve therapeutic complement inhibition, we have to recognize that discontinuation of current treatment may have catastrophic impact on patient outcomes and must to be avoided. In this context, it is clear that the positive impact of eculizumab on survival is largely due to the prevention of thromboembolic events, which is not necessarily mechanistically associated with hematological response. Thus, even if we can postulate that novel, more effective strategies of complement inhibition will have similar impact on thromboembolism and thus on survival, this will have to be demonstrated in long-term studies. Further investigations are also needed to better understand the reasons underlying “breakthrough thromboembolisms” occurring during eculizumab treatment, which could be associated with suboptimal complement blockade, or being independent of PNH: in the former case, novel strategies of complement inhibition might have a therapeutic role.

### A More Effective C5-Inhibition Is Possible

For more than a decade we have thought that C5 blockade by eculizumab (at standard doses) was the perfect C5 blockade, with the exception of patients carrying the R885H C5 polymorphism; long-term clinical outcome is outstanding, (23, 24) and even persistent, mildly increased LDH is clinically irrelevant (42). However, looking to recent data coming from new anti-C5 agents, it is obvious that therapeutic C5 blockade can be improved not only in terms of patient comfort (e.g., administration route, interval between administrations, self-administration). Recent PK/PD data coming from comparison between ALXN1210 and eculizumab in untreated PNH patients (86) (thus, no selection bias based on previous response to eculizumab) show that free C5 (a PK biomarker rather than a PD one, since it reflects anti-C5 mAb concentration in relation with C5 level, instead of C5 cleavage, which is the activity inhibited by anti-C5 mAbs) consistently remains below the threshold of 0.5 μg/mL (about 0.5% of normal C5 plasma level) throughout the 6-month treatment period with ALXN1210, whereas 12.4% of patients treated with eculizumab at the dose of 900 mg every other week exhibited free C5 above this threshold at the time of some administration during the treatment (132). These data support the clinical observation that about 15% of PNH patients are under dosed with the current approved dose of eculizumab (21, 42, 46), while the treatment schedule developed for ALXN1210 (through a formal dose-finding study) (84) eventually results in a “deeper” (or should we say “better sustained”) C5 blockade (132). However, this prevention of transient loss of C5 blockade does not result in evident clinical benefit, since no difference even in terms of LDH levels were seen between ALXN1210 and eculizumab (87, 89). Interestingly, even if the rarity of the event precludes robust conclusions, breakthrough episodes seem to be reduced in ALXN1210 as compared to eculizumab: there were five episodes in the ALXN1210 arm vs. 15 in the eculizumab arm (158). Notably, in the eculizumab arm breakthrough episodes were often (7/15) associated with free C5 >0.5 μg/mL, whereas this was never found in the ALXN1210 arm (158). This observation supports the distinction between PK and PD breakthrough intravascular hemolysis (Table 4): the former is always associated with free C5 >0.5 μg/mL due to inadequate plasma level of anti-C5, and can easily be prevented by changing the treatment schedule of eculizumab (21, 42, 46). In contrast, PD breakthrough intravascular hemolysis (e.g., infection-associated triggering massive complement activation) may develop even during more effective therapeutic C5 inhibition, at least with anti-C5 mAb (e.g., increased dose of eculizumab, or standard dose of ALXN1210), and seems to not benefit from any intervention on anti-C5 treatment schedule. Finally, we have to acknowledge that, pharmacodynamically speaking, C5 blockade may be even more effective: indeed, when anti-C5 mAb (eculizumab) was combined with an anti-C5 si-RNA the desired LDH normalization was seen (109); perhaps in these circumstances even the excess of high-affinity C5 convertases (57) could not overcome the complete lack of substrate. It remains an open question whether this better control of intravascular hemolysis will be clinically meaningful.

### Proximal Complement Inhibition Is Feasible

Inhibitors of the proximal complement have been specifically designed to address the emerging problem of C3-mediated extravascular hemolysis (Figure 1) (28, 64, 66); at the moment, they include agents targeting either C3, complement FD and complement FB. Their clinical development was complicated by the concern about a possible increased risk of infectious and auto-immune complications, but this so far has not been observed in studies. Data are obviously less mature as compared with those of anti-C5 agents, but, as anticipated by their distinct mechanism of action, a more profound clinical benefit may be achieved with these newer agents. Indeed, novel in PNH, the use of therapies able to prevent both intravascular and extravascular hemolysis may lead to major improvement in the resolution of anemia. Moreover, the proof that the pathogenesis of PNH (as a hemolytic disorder) is completely disabled comes from the observation that, in addition to hemoglobin normalization: (i) any sign of hemolysis disappears (normal bilirubin and haptoglobin); (ii) no compensatory erythropoiesis is detected (reticulocyte count normalizes); (iii) life-span of PNH erythrocytes is close to normal (the size of PNH erythrocyte population increases close to that of granulocytes). Not all proximal complement inhibitors are the same, but since data are still preliminary at this stage we cannot dissect the therapeutic efficacy of these different strategies thus far; however, some speculations can be done. Anti-FD and anti-FB agents have the obvious advantage of oral administration (even if the short half-life requires 2–3 administrations per day), which is possibly counterbalanced by the fact that they inhibit only the alternative pathway of complement, that anyhow also serves to amplify the activation triggered through the classical or the mannose/lectin pathways. We can speculate that C3 inhibitors are more likely to achieve the best results even as monotherapy, since they disable all complement pathways. Indeed, if therapeutic C3 inhibition is pharmacodynamically complete, and the PK profile allows a treatment schedule not associated with transient sub-therapeutic plasma levels of the agent, breakthrough hemolysis is not expected; in this case, the concomitant treatment with anti-C5 treatment would be unnecessary. On the other hand, proximal inhibitors of the complement alternative pathway (i.e., anti-FD and anti-FB) might fail in fully preventing hemolysis if complement is activated through the classical or the mannose/lectin pathways (which might be the case of infection-driven hemolysis), even if the inhibition of the alternative-pathway mediated amplification loop may result in substantial reduction of hemolysis in these conditions. Thus, with anti-FD and anti-FB inhibitors, it will have to be investigated whether the highest hematological response expected when they are used in combination with an anti-C5 agent will be retained also in monotherapy. While this conservative approach appears initially appropriate in the interest of patients, it is also possible that, since the alternative pathway is the key pathogenic mechanism of PNH, the addition of an anti-C5 agent is not needed with anti-FD and anti-FB. It is important to acknowledge that, due to the significant increase of PNH erythrocyte mass associated with the prevention of C3-mediated extravascular hemolysis, rebound breakthrough hemolysis may be a concern during treatment with proximal complement inhibitors. This risk needs to be mitigated by treatment schedules which prevent any decrease of complement inhibition even in case of missing doses (especially when frequent dosing is used, and self-administration increases the risk of inadequate compliance).

### Clinical Endpoints Need to be Redefined

In a chronic, life-threatening disease such as PNH short-term surrogate endpoints are needed for clinical trials. In the eculizumab era, when PNH patients were heavily transfused, transfusion avoidance and hemoglobin stabilization were obvious goals which have been achieved, together with reduction of LDH (which served as a biomarker of disease activity rather than as an endpoint *per se*). Now, it is a matter of debate in identifying the clinically meaningful endpoints that can be exploited in future trials investigating novel anti-complement agents. Logically, LDH is an obvious choice, since it is a marker of intravascular hemolysis (i.e., of disease activity) and it remains slightly increased even during effective eculizumab treatment. Many new anti-C5 agents were designed to deliver more effective C5 blockade, and changes in LDH was used as primary endpoint in their trials; however, it remains debatable if any reduction in LDH (e.g., from the median 1.5 times the ULN seen with eculizumab, to normal levels) by itself may be considered a clinically meaningful endpoint. The frequency of breakthrough hemolysis is another endpoint that has been exploited, but it appears specifically linked to suboptimal dosing of eculizumab; indeed, at least PK breakthrough should not be a problem with novel anti-C5 agents, while PD breakthrough remains possible (at least with anti-C5 agents used in monotherapy). Likely, more robust goals are needed, and the hemoglobin level appears a more proper endpoint given its intimate association with clinical manifestations and a consequence of all the pathogenic events underlying PNH (intravascular hemolysis, extravascular hemolysis and bone marrow function). Notably, improvement of anemia was not the only benefit of eculizumab treatment, since the most important effect impacting long-term survival was the reduction of thromboembolic events (22). Unfortunately, the time-frame to assess the thromboembolic risk is extremely long, and surrogate endpoints would be welcome. At the moment there is no evidence concerning the possible relationship between low-level residual intravascular hemolysis or breakthrough hemolysis and thromboembolic risk, but it is conceivable that a more effective inhibition of the terminal complement pathway (and possibly of the whole complement cascade in general) might have an effect also on thrombophilia of PNH (159). Nevertheless, despite this considerable scientific background, caution should be used and the efficacy of newer anti-complement agents on prevention and treatment of PNH-related thromboembolism needs to be carefully investigated, since it will obviously impact long-term morbidity and mortality.

### Toxicity of Standard and Novel Complement Inhibition

Toxicity remains a major concern for any novel treatment, especially in a disease with such a good long-term outcome as PNH treated with eculizumab. Historically, the development of anti-complement treatment raised several concerns about the risk of infectious complications. Indeed, after cases of infection by *N. meningitidis* seen in initial phases of clinical development of eculizumab (still in indications other than PNH, such as auto-immune diseases), vaccination against this microorganism was mandatory for all patients prior to initiating C5 blocking therapy. Data from post-marketing pharmacovigilance did not lead to any alert concerning the risk of meningococcal infection, nor of any other life-threatening infection (160). Indeed, after more than 10 years of experience with eculizumab it appears evident that the initial concern of infectious toxicity has been largely mitigated, also as a result of prophylactic measures routinely employed in PNH patients on eculizumab. Theoretically, most anti-C5 agents will recapitulate the safety profile seen with eculizumab; however, we must acknowledge that a more effective C5 blockade (which appears to be a clinical goal) might also result in increased risk of infectious complications. In other words, while the low-level residual C5 activity (as demonstrated by slightly increased LDH) seen on eculizumab may be detrimental for hematological benefit, it might be sufficient to eradicate microorganisms at time of infections. Notably, in considering the molecular mechanisms, this low-level residual C5 activity may be due to high-affinity C5 convertases (57) generated on bacteria, which would displace free C5 from eculizumab similarly to what we have described for PD breakthrough. In moving from a more effective C5 blockade to a blockade of the proximal complement the discussion becomes even more complicated: these agents were designed to completely shut down complement (to prevent any residual hemolysis of PNH erythrocytes), but this may eventually lead to higher risk of infectious complications. Some insight may be obtained by analyzing rare families carrying genetic deficiencies of these complement components (161–164). Subjects with inherited C3 deficiency (about 20 families described so far) seem to have an increased risk of infections by encapsulated bacteria (e.g., *N. meningitidis, S. pneumoniae*, and *H. influenzae*), which tend to be severe and recurrent (161–164), as well as some risk of autoimmune diseases (165). Recurrent infections have been described also in very rare subjects with inherited deficiencies of properdin (166), complement FD (167), and complement FB (168) even if in this case the clearance of infectious agents may be addressed by the classical pathway, as demonstrated *in vitro* (169). However, all these infections occur usually in childhood, and tend to become infrequent when adaptive immunity has established. These observations, together with the consideration that therapeutic inhibition is not a phenocopy of inherited deficiencies (i.e., the role of complement in the development of innate immunity is fully preserved), support the concept that therapeutic inhibition of proximal complement should be feasible (170). This assumption seems confirmed by recently available data from systemic C3 inhibition, which was not associated with any increased infectious risk, at least with a conservative risk-mitigation strategy based on strict prophylactic measures (117). Nevertheless, the safety of each of these newer agents will require careful monitoring, and possible off-target effects (including any detrimental effect on the bone marrow function) need to be ruled out.

### And the Winner Is …

The definition of the best drug for PNH treatment is timely; however, we must think about the best strategy rather than about the best drug. Of course, companies are motivated to demonstrate that one drug is better than another, but this is not necessarily useful for patients: we first need to better understand what is the greatest clinical benefit that may be achieved in PNH patients. We have already discussed available results with each of these novel anti-complement agents, together with their mechanistic goals; interestingly, some agents are used both in monotherapy and in combination with standard anti-C5 treatment, emphasizing that clinical data are essential to prove the initial hypothesis. Novel strategies of C5 inhibition definitely address some patients' needs, such as possible self-administration or extending the interval between IV dosing; however, it is likely that they will not lead to superior clinical benefit, except better patients' convenience. Some novel anti-C5 agents might deliver a more effective C5 blockade, but the actual benefit to patients of a further LDH reduction is uncertain. Indeed, even the best C5 inhibition seen with the combination of eculizumab with the anti-C5 si-RNA will control only residual intravascular hemolysis, with a hematological benefit that for the majority of patients is minor. It is most likely that the next breakthrough in PNH treatment will come from the inhibitors of the proximal complement pathway: anti-C3, anti-FD and anti-FB agents. Preliminary data clearly demonstrate that by interfering with the complement cascade upstream they inhibit MAC-mediated intravascular hemolysis and prevent C3-mediated extravascular hemolysis; but how profound is the inhibition of these targets is unclear. The lesson from the anti-C5 was very instructive: even minimal residual amounts of these complement proteins may be enough to keep complement activity almost intact, likely because they are substrates or very active enzymes generated at time of complement activation. Proximal inhibitors appear to disable all disease mechanisms in hemolysis of PNH; however, we still don't know whether inhibition of C3 is pharmacologically effective enough to prevent possible residual activity (as demonstrated for C5, for instance for PD breakthrough), and whether their targets in the complement cascade may be somehow by-passed in specific clinical circumstances (e.g., for anti-FD and anti-FB in case of complement activation through the classical and mannose/lectin pathways; and for anti-FD also in case of C3 activation by other plasma protease, i.e. FD by-pass) (171). This information is essential to understand whether proximal complement inhibitors can be used as monotherapy, or whether a combination with an anti-C5 agent is needed to achieve a better hematological response. Indeed, the hypothesis of a combined treatment with anti-C5 and proximal inhibitors is also supported by *in vitro* data showing synergy between eculizumab and anti-FD (172). These data may raise the hypothesis that a less than optimal inhibition of two different steps of the complement cascade (e.g., an anti-C5 agent combined with a proximal inhibitor) may result in similar hematological benefit, with mitigation of the infectious risk due to residual, low-level “protective” complement activity.

Thus, we envision a new scenario in the treatment of PNH where inhibitors of the proximal complement (either anti-C3, anti-FD or anti-FB) are essential, and if pharmacologically adequate may be used even in monotherapy to control intravascular and extravascular hemolysis. Alternatively, these proximal inhibitors might require use in combination with anti-C5 agents, possibly long-acting, to maximize therapeutic efficacy. In this scenario, anemia from both intravascular and extravascular hemolysis would be fully prevented, and normal hemoglobin is expected in absence of bone marrow failure. In this new scenario, we must not forget that the future pricing of these new agents remains a major issue. Unfortunately, national pricing scheme policies on orphan medicine products has allowed exaggerated prices, which seem not always justified by their cost (i.e., manufacturing, research, and development) and their actual clinical value (i.e., impact on life expectancy and quality of life) (82). Indeed, in PNH the very high price of the only approved drug eculizumab has hindered its use in many countries, and even in countries where it is available its prescription was restricted to PNH patients with more symptomatic disease (i.e., severe anemia, or symptomatic hemolysis or life-threatening thrombosis). While the goal of very high profit has driven the interest of pharmaceutical companies, now we hope that this competition will lead to a considerable reduction of drug price. This remains a major need in a world-wide scientific/medical community, since, from the point of view of scientists and physicians working in the field, one additional goal of these exciting developments is to provide effective drugs with reasonable prices, allowing a broad use of effective treatments worldwide.

## Conclusions

In the last decade we have been able to offer PNH patients an almost-normal life-expectancy, irrespective of their disease (23, 24): this was a terrific milestone in medicine. Nevertheless, new challenges and goals are coming, and we are starting to wonder what's in store for PNH patients in the next decade. Thanks to the first therapeutic C5 inhibitor eculizumab we have controlled the most debilitating symptoms of PNH, and prevented the most devastating life-threatening complications, such as thromboembolism. Perspectives of further improvements seem within reach with the introduction of therapies which are less burdensome for patients and may act on their perception of illness: indeed, very long intervals of dosing and/or self-administration of second generation complement inhibitors may help patients become less challenged by their disease. Shall we go beyond this? We think so, and we feel now ready for a real breakthrough in the field coming with the introduction of proximal complement inhibitors. Perhaps the time when PNH patients may live with their aberrant blood cells while keeping normal hemoglobin values and having no signs or symptoms of disease is not too far away. In the next few years this will represent a major achievement in the field, while in the long-term we will continue to pursue our goal of a definitive cure for PNH.

## Author Contributions

AR, RN, and RP conceived the study and identified the other experts who contributed to the generation of the consensus. AR, AK, RC, PS, RN, and RP wrote the manuscript and together with the other SM, PR, LM, CF, FC, and MS generated the consensus on all the topics discussed in the manuscript. All the authors have critically revised the manuscript and contributed to its preparation in the current version.

### Conflict of Interest Statement

AR has received research support from Alexion, Novartis, Alnylam and Rapharma, lecture fees from Alexion, Novartis, Pfizer and Apellis, and served as member of advisory/investigator board for Alexion, Roche, Achillion, Novartis, Apellis and Samsung, and served as consultant for Amyndas. RP has received research funding from Alexion, Amgen, Jazz Pharmaceuticals and Pfizer; consulted for, and received honoraria from Alexion, Amgen, Gilead, Jazz Pharmaceuticals, Keocyte, MSD, Novartis, Pfizer, Roche, Samsung and Mallinckrodt. The remaining authors declare that the research was conducted in the absence of any commercial or financial relationships that could be construed as a potential conflict of interest.

## Footnotes

^1^https://www.samsungbioepis.com/en/product/product02.do
